# Detection and Estimation of Diffuse Signal Components Using the Periodogram

**DOI:** 10.3390/s24206650

**Published:** 2024-10-15

**Authors:** Jesus Selva

**Affiliations:** Department of Physics, Systems Engineering and Signal Theory (DFISTS), University of Alicante, P.O. Box 99, E-03080 Alicante, Spain; jesus.selva@ua.es

**Keywords:** periodogram, beamformer, maximum likelihood estimation, diffuse components, discrete Chebyshev polynomials, frequency estimation, channel estimation

## Abstract

One basic limitation of using the periodogram as a frequency estimator is that any of its significant peaks may result from a diffuse (or spread) frequency component rather than a pure one. Diffuse components are common in applications such as channel estimation, in which a given periodogram peak reveals the presence of a complex multipath distribution (unresolvable propagation paths or diffuse scattering, for example). We present a method to detect the presence of a diffuse component in a given peak based on analyzing the projection of the data vector onto the span of the signature’s derivatives up to a given order. Fundamentally, a diffuse component is detected if the energy in the derivatives’ subspace is too high at the peak’s frequency, and its spread is estimated as the ratio between this last energy and the peak’s energy. The method is based on exploiting the signature’s Vandermonde structure through the properties of discrete Chebyshev polynomials. We also present an efficient numerical procedure for computing the data component in the derivatives’ span based on barycentric interpolation. The paper contains a numerical assessment of the proposed estimator and detector.

## 1. Introduction

The periodogram of a finite data sequence consists of the square absolute value of its Discrete-Time Fourier Transform (DTFT) and is one of the most versatile tools in Signal Processing. Just to note a few of its uses, it is a well-known power spectral-density estimator and the basis of more elaborate estimators of the same type ([1] Ch. 6). It allows us to implement the Maximum Likelihood (ML) estimator of a single frequency and a high-quality estimator for multiple well-separated frequencies ([2] Sec. 13.3.2), [3,4]. In addition, it is also applicable to delays and angles of arrivals, given that these parameter types can be turned into frequencies either by means of the Fourier Transform or by exploiting a uniform and linear array geometry, respectively [5,6]. Finally, it also provides channel impulse–response estimates [7,8].

The periodogram can be efficiently sampled using the Fast Fourier Transform (FFT), a fact that seems to be the main factor behind its popularity. Actually, this sampling just involves O(NlogN) operations, where *N* is the data sequence length. Extensive literature exists on methods for estimating multiple frequencies that complement this FFT sampling with other operations, such as the computation of additional DTFT samples or the evaluation of interpolators [9,10,11,12,13,14,15,16,17]. These additional operations have complexity O(NK), where *K* is the number of frequency estimates, except for the method in [12,15] in which their complexity is O(K); (see also [18] for the interpolation method used in these last references).

In most cases, frequency estimation using the periodogram is carried out assuming well-separated pure components, i.e., the data is assumed to consist of a sum of K>0 components in white noise. However, in channel estimation, this assumption is often inadequate, given that physical phenomena such as reflections on rough surfaces or multiple closely spaced specular reflections produce diffuse or spread components that appear as a single peak in the periodogram [8,19,20,21,22]. Intuitively, we may expect the shape of a given periodogram peak to reveal, in some way, whether it is produced by a diffuse or a pure component. In this paper, we specify this intuition by presenting a method to detect whether a given periodogram peak is diffuse and to estimate its “spreadness”, i.e., the degree to which it is diffuse. The method is based on analyzing the subspace spanned by the signature’s derivatives (up to a given order) at the peak’s frequency.

The paper has been organized as follows. In the next section, we present the signal models for a pure and a diffuse component, the periodogram function, and sketch the proposed method for the detection and estimation of diffuse components. Then, in Section 3, we derive the Gram–Schmidt basis associated with the frequency signature and its derivatives, which is the main analytical tool in subsequent sections. After that, we obtain the statistical distribution of the projection of the data vector onto the span of the signature’s derivatives in Section 4, and we present the proposed estimator and detector in Section 5. Section 6 is dedicated to an efficient computation method for the correlation samples required by the method which is based on barycentric interpolation. Finally, we assess the proposed detector and estimator in Section 7 numerically.

### Notation

The notation in the paper is as follows:

- We denote column vectors in bold and lower case (x, a).
- $I_{N}$ denotes the N×N identity matrix.
- ${\left[a\right]}_{n}$ denotes the *n*th component of vector a.
- $x^{T}$ and $x^{H}$ are the transpose and Hermitian of vector x, respectively.
- New symbols or functions are introduced using the symbol “≡”.
- $\delta_{p}$ is the discrete Dirac delta, i.e., $\delta_{0}=1$ and $\delta_{p}=0$ for integer p≠0.
- 0 denotes a column vector of zeroes whose length can be determined from the context.
- For a function g(ϵ), $dg_{\epsilon_{o}}\left(\gamma \right)$ denotes the total derivative of g(ϵ) at the value $\epsilon =\epsilon_{o}$, i.e., $dg_{\epsilon_{o}}\left(\gamma \right)$ is the best linear approximation to g(ϵ) at $\epsilon =\epsilon_{o}$.

## 2. Signal Models for a Pure and a Diffuse Component: Sketch of the Proposed Method

Consider a N×1 data vector x consisting of a single undamped exponential of frequency $f_{o}$ in noise,

(1) $$x=a\left(f_{o}\right)s_{o}+\epsilon ,$$

where

(2) $${\left[a\left(f\right)\right]}_{n+1}\equiv e^{j2\pi nf},$$

$s_{o}$ is a complex amplitude, and ϵ an N×1 complex zero-mean circularly symmetric white noise vector of variance $\sigma^{2}$. The Deterministic Maximum Likelihood (DML) estimate of $f_{o}$ in (1) is the abscissa $\hat{f}\left(x\right)$ at which the global maximum of the periodogram function

(3) $$P\left(f;x\right)\equiv {\left|a{\left(f\right)}^{H}x\right|}^{2}$$

is attained, that is,

(4) $$\hat{f}\left(x\right)\equiv \arg\max_{f}P\left(f;x\right).$$

(See ([3] Sec 3.B)). This frequency estimator is popular due to its statistical efficiency and because it can be efficiently computed through an FFT algorithm followed by a numerical method [9,15].

Equation (1) is often a simplified model for a situation in which there is actually some frequency spread along a short band [a,b]. For instance, in channel sounding, the periodogram’s peak may be produced by an imperfect specular reflection, i.e., a reflection on an irregular surface, and a more realistic model would be

(5) $$x=\int_{a}^{b}a\left(f\right)s\left(f\right)df+\epsilon$$

for a function s(f). Here, s(f) is completely unknown and hardly estimable in practice. It can be, for example, a sum of $K^{\prime }>1$ components, i.e.,

(6) $$s\left(f\right)=\sum_{k=1}^{K^{\prime }}s_{k}\delta \left(f-f_{k}\right)$$

for complex coefficients $s_{k}$ and frequencies $f_{k}$, $a\leq f_{k}\leq b$; a continuous function in [a,b]; or any distribution we can think of in [a,b].

Assume now that the periodogram has a significant peak at frequency $\hat{f}$ for which both (1) and (5) seem plausible. The question is whether there is a detector that allows us to select either (1) or (5). In this detector, (1) would be hypothesis H0 and (5) hypothesis H1. To sketch a possible detector, consider (1) and (5) without noise (ϵ=0). In this case, the periodogram estimate is exact assuming (1), that is, $\hat{f}\left(x\right)=f_{o}$, and x lies in the span of $a(\hat{f}\left(x\right))$, given that

(7) $$x=a\left(f_{o}\right)s_{o}=a\left(\hat{f}\left(x\right)\right)s_{o}.$$

However, assuming (5), $\hat{f}\left(x\right)$ turns out to be a frequency in [a,b] and x fails to lie in the span of $a(\hat{f}\left(x\right))$. But since [a,b] is short, a truncated Taylor expansion of small order *P*, such as

(8) $$a\left(f\right)\approx \sum_{p=0}^{P}a^{\left(p\right)}\left(\hat{f}\left(x\right)\right)\frac{{\left(f-\hat{f}\left(x\right)\right)}^{p}}{p!}$$

is accurate in [a,b] and, therefore, x approximately lies in the span of $a^{\left(0\right)}\left(\hat{f}\right)$, $a^{\left(1\right)}\left(\hat{f}\right),\;\ldots ,$ $a^{\left(P-1\right)}\left(\hat{f}\right)$, given that

(9) $$\begin{array}{cc} x & \approx \int_{a}^{b}\sum_{p=0}^{P}a^{\left(p\right)}\left(\hat{f}\left(x\right)\right)\frac{{\left(f-\hat{f}\left(x\right)\right)}^{p}}{p!}s\left(f\right)df=\sum_{p=0}^{P}a^{\left(p\right)}\left(\hat{f}\left(x\right)\right)\int_{a}^{b}\frac{{\left(f-\hat{f}\left(x\right)\right)}^{p}}{p!}s\left(f\right)df. \end{array}$$

Thus, if we decompose x in two components

(10) $$x=\hat{x}+{\hat{x}}^{⊥},\;\;\left({\hat{x}}^{H}{\hat{x}}^{⊥}=0\right),$$

where $\hat{x}$ lies in the span of $a^{\left(0\right)}(\hat{f})$ and ${\hat{x}}^{⊥}$ lies in the orthogonal complement of $a^{\left(0\right)}\left(\hat{f}\right)$ relative to the span of $a^{\left(0\right)}\left(\hat{f}\right),\;a^{\left(1\right)}\left(\hat{f}\right),\;\ldots ,\;a^{\left(P\right)}\left(\hat{f}\right)$, then we have a diffuse component that can be detected by a non-zero vector ${\hat{x}}^{⊥}$.

In order to carry over this idea to the noisy case (ϵ≠0), we would need to find out the statistical distribution of ${\hat{x}}^{⊥}$ under model (1), and then select a threshold γ, such that ${∥{\hat{x}}^{⊥}∥}^{2}>\gamma$ implies that model (1) must be discarded and (5) accepted with a given false-alarm probability $P_{\mathrm{FA}}$. We can simplify this task significantly if we start by describing the span of $a^{\left(0\right)}\left(\hat{f}\right),\;a^{\left(1\right)}\left(\hat{f}\right),\;\ldots ,\;a^{\left(P\right)}\left(\hat{f}\right)$ in terms of a Gram–Schmidt basis. More precisely, let $\phi_{0}\left(f\right)$, $\phi_{1}\left(f\right),\;\ldots$, $\phi_{P}\left(f\right)$ denote a set of N×1 signatures, such that

- $\phi_{p}{\left(f\right)}^{H}\phi_{q}\left(f\right)=\delta_{p-q}$; 0≤p≤P, 0≤q≤P.
- $\left[\phi_{0}\left(f\right),\;\ldots ,\;\phi_{p}\left(f\right)\right]$ has the same span as $[a_{0}\left(f\right),\;\ldots ,$ $a_{p}\left(f\right)]$ for p=0, 1,…, *P*.

In terms of this basis, ${\hat{x}}^{⊥}$ can be written as

(11) $${\hat{x}}^{⊥}=\sum_{p=1}^{P}\phi_{p}\left(\hat{f}\right)\phi_{p}{\left(\hat{f}\right)}^{H}x$$

and the test’s inequality would be

(12) $${∥x^{⊥}∥}^{2}=\sum_{p=1}^{P}{\left|\phi_{p}{\left(\hat{f}\right)}^{H}x\right|}^{2}<\gamma .$$

We carry out this plan in the next three sections:

- We derive the ortho-normal basis $\phi_{0}\left(f\right)$, $\phi_{1}\left(f\right),\;\ldots$, $\phi_{P}\left(f\right)$ in Section 3. The main tool for this is the set of discrete Chebyshev polynomials ([23] p. 33).
- We obtain the statistical distribution of the correlations $\phi_{p}{\left(\hat{f}\right)}^{H}x$ and p=0,1,…,P in Section 4 by means of a perturbation analysis of the periodogram estimate $\hat{f}$.
- We present the proposed detector and estimator in Section 5.

Finally, regarding the case of a periodogram with K>1 significant peaks, it is straightforward that (1) and (5) can be extended to the models

(13) $$x=\sum_{k=1}^{K}a\left(f_{o,k}\right)s_{o,k}+\epsilon$$

and

(14) $$x=\sum_{k=1}^{K}\int_{a_{k}}^{b_{k}}a\left(f\right)s\left(f\right)df+\epsilon$$

where $f_{o,k}$ are separate frequencies and $\left[a_{k},b_{k}\right]$ disjoint intervals containing the diffuse components, k=1,2,…,K. The argument just presented for (5) is applicable to any of the *K* periodogram peaks, say the *k*th, if its corresponding interval $\left[a_{k},b_{k}\right]$ is sufficiently separated from the other peaks’ intervals. However, if the separation with, say, component k+1 is small, then there can be a significant contribution of component k+1 to the orthogonal component ${\hat{x}}^{⊥}$ of the *k*th peak. We do not discuss this more complex case in this paper, though we can envisage two possible strategies for dealing with it: we can either increase the threshold γ in (12) in terms of the separation between $\left[a_{k},b_{k}\right]$ and any adjacent peak interval, or we can apply some kind of windowing in order to select just one periodogram peak. This second possibility appears naturally in the proposed method, as shown in Section 3.3.

## 3. Gram–Schmidt Basis of the Span of the Signature and Its Derivatives

In the sequel, we present the Gram–Schmidt basis $\phi_{0}\left(f\right),$$\phi_{1}\left(f\right),\;\ldots ,$$\phi_{N-1}\left(f\right)$ in three separate sub-sections. In the first, we define it in terms of the so-called discrete Chebyshev polynomials. Then, in Section 3.2, we derive a key property of this basis, namely, the fact that the derivative of any $\phi_{p}\left(f\right)$ belongs to the span of three consecutive signatures $\phi_{p}\left(f\right)$ at most. This property is fundamental in the results presented in Section 4. Finally, we show in Section 3.3 that we can select or “filter” the signatures with frequencies inside a range of variable length by truncating the basis. This feature is useful given that it allows us to employ the model in (5) for one diffuse peak, even if there are several significant periodogram peaks.

### 3.1. Definition of the Gram–Schmidt Basis in Terms of Discrete Chebyshev Polynomials

In order to derive the ortho-normal basis $\phi_{0}\left(f\right)$, $\phi_{1}\left(f\right),\;\ldots$, $\phi_{P}\left(f\right)$, consider the following formula for the derivatives of a(f),

(15) $$a^{\left(p\right)}\left(f\right)={\left(j2\pi \right)}^{p}\mathrm{diag}\left(a\left(f\right)\right)v_{p}$$

where $v_{p}$ is the N×1 Vandermonde vector

(16) $${\left[v_{p}\right]}_{n+1}\equiv n^{p},\;\;(\mathrm{taking}\;0^{0}=1).$$

In (15), since ${\left(j2\pi \right)}^{p}$ is a constant and diag(a(f)) a unitary transformation, it follows that any ortho-normal basis $y_{0}$, $y_{1},\;\ldots$, $y_{P}$ of the span of $v_{0}$, $v_{1},\;\ldots$, $v_{P}$ produces an ortho-normal basis of the span of $a^{\left(0\right)}\left(f\right),\;a^{\left(1\right)}\left(f\right),\;\ldots ,\;a^{\left(P\right)}\left(f\right)$ given by the signatures

(17) $$\phi_{p}\left(f\right)\equiv \mathrm{diag}\left(a\left(f\right)\right)y_{p},\;\;p=0,\;1,\;\ldots ,\;P.$$

A basis $y_{0}$, $y_{1},\;\ldots$, $y_{P}$ can be obtained through the Gram–Schmidt process and, additionally, its vectors $y_{p}$ can be expressed in closed form if they are related with the so-called discrete Chebyshev polynomials ([24] p. 58).

Let $\pi_{N-1}$ denote the set of real polynomials of order at most N−1 equipped with the inner product

(18) $$\langle a,b\rangle \equiv \sum_{n=0}^{N-1}a\left(n\right)b\left(n\right);\;\;a,b\in \pi_{N-1}.$$

Given a polynomial $z\in \pi_{N-1}$, the sampling operator S{·} defined by

(19) $$S\left\{z\right\}\equiv {\left[z\left(0\right),z\left(1\right),\;\ldots ,\;z\left(N-1\right)\right]}^{T}$$

assigns to *z* its vector of samples at 0,1,…,N−1. Additionally, S{·} has an inverse, which is the interpolation operator I{·} that assigns to any vector $w\in R^{N}$ the unique polynomial $I\left\{w\right\}\in \pi_{N-1}$, such that $I\left\{w\right\}\left(n\right)={\left[w\right]}_{n+1}$, n=0,1,…,N−1. It can be readily checked that I is an isomorphism mapping the inner-product space $R^{N}$ onto the inner-product space $\pi_{N-1}$. This implies that the problem of ortho-normalizing $v_{0},v_{1},\;\ldots ,\;v_{P}$ is equivalent to that of ortho-normalizing the image of these vectors in $\pi_{N-1}$, which is the set of monomials $I\left\{v_{p}\right\}\left(n\right)=n^{p}$, p=0,1,…,P. The discrete Chebyshev polynomials is the unique Gram–Schmidt basis (up to scale factors) for this last set for any *P*. Specifically, the *p*-th order polynomial of this kind $\alpha_{p}\left(n\right)$ is defined by the recurrence

(20) $$\begin{array}{cc} \alpha_{0}\left(n\right) & \equiv 1\\ \alpha_{1}\left(n\right) & \equiv N-1-2n\\ \alpha_{p}\left(n\right) & \equiv \frac{\left(2p-1)(N-1-2n\right)}{p^{2}}\alpha_{p-1}\left(n\right)-\frac{N^{2}-{\left(p-1\right)}^{2}}{p^{2}}\alpha_{p-2}\left(n\right),\;\;p>1. \end{array}$$

If P<N, the first P+1 polynomials $\alpha_{p}\left(n\right)$ form an orthogonal basis of the span of $n^{p}$, p=0,1,…,P. Thus, if we define from them a normalized set of polynomials, given by

(21) $$y_{p}\left(n\right)\equiv \frac{\alpha_{p}\left(n\right)}{∥\alpha_{p}∥},\;\;p=0,\;1,\;\ldots ,\;P,$$

where for any $z\in \pi_{N-1}$ it is $∥z∥\equiv \sqrt{\langle z,z\rangle }$, we have that the polynomials $y_{p}$ form an ortho-normal basis of the span of $n^{p}$, p=0,1,…,P. Additionally, $y_{p}\left(n\right)$ has order *p*, i.e., there is a set of coefficients $\psi_{p,q}$, such that

(22) $$y_{p}\left(n\right)=\sum_{q=0}^{p}\psi_{p,q}n^{q},\;p=0,\;1,\;\ldots ,P.$$

The desired basis $y_{0}$, $y_{1},\;\ldots$, $y_{P}$ is the result of sampling the polynomials $y_{p}\left(n\right)$, noting that $n^{p}={\left[v_{p}\right]}_{n+1}$,

(23) $$y_{p}\equiv S\left\{y_{p}\right\}=\sum_{q=0}^{p}\psi_{p,q}v_{q},\;\;p=0,\;1,\;\ldots ,\;P.$$

The first three $y_{p}$ are the following, (n=0,1,…,N−1):

(24) $$\begin{array}{cc} {\left[y_{0}\right]}_{n+1} & \equiv \frac{1}{\sqrt{N}} \end{array}$$

(25) $$\begin{array}{cc} {\left[y_{1}\right]}_{n+1} & \equiv \frac{\sqrt{3}\left(N-2n-1\right)}{\sqrt{N(N^{2}-1)}} \end{array}$$

(26) $$\begin{array}{cc} {\left[y_{2}\right]}_{n+1} & \equiv \frac{\sqrt{5}\left(N^{2}-6nN-3N+6n^{2}+6n+2\right)}{\sqrt{N(N^{4}-5N^{2}+4)}}. \end{array}$$

Finally, recalling (15) and (17), we may write $\phi_{p}\left(f\right)$ as a linear combination of $a^{\left(q\right)}\left(f\right)$, q=0,1,…, *p*,

(27) $$\begin{array}{c} \phi_{p}\left(f\right)=\mathrm{diag}\left(a\left(f\right)\right)y_{p}=\mathrm{diag}\left(a\left(f\right)\right)\sum_{q=0}^{p}\psi_{p,q}v_{q}=\sum_{q=0}^{p}\frac{\psi_{p,q}}{{\left(j2\pi \right)}^{q}}a^{\left(q\right)}\left(f\right),\;\;p=0,\;1,\;\ldots ,\;P. \end{array}$$

### 3.2. Three-Term Relationship for Basis Signature Derivatives

Let us take (27) as the definition of the ortho-normal basis of the span of $a^{\left(0\right)}\left(f\right)$, $a^{\left(1\right)}\left(f\right),\;\ldots$, $a^{\left(P\right)}\left(f\right)$. This basis has a property, stemming from the three-term recurrence Formula (20), that is key in the next sections. The property is that the derivative of any of the basis vectors $\phi_{q}^{\prime }\left(f\right)$ lies in the span of at most the three signatures $\phi_{q-1}\left(f\right)$, $\phi_{q}\left(f\right)$ and $\phi_{q+1}\left(f\right)$, (excluding $\phi_{q-1}\left(f\right)$ if q=0 and $\phi_{q+1}\left(f\right)$ if q=N−1). Specifically, since the set $\phi_{0}\left(f\right)$, $\phi_{1}\left(f\right),\;\ldots ,$$\phi_{N-1}\left(f\right)$ forms an ortho-normal basis of $C^{N}$, $\phi_{q}^{\prime }\left(f\right)$ can be expressed in this last basis using a set of coefficients $h_{p,q}\left(f\right)$, i.e., it is

(28) $$\phi_{q}^{\prime }\left(f\right)=j\sum_{p=0}^{N-1}h_{p,q}\left(f\right)\phi_{p}\left(f\right).$$

We prove in Appendix A that the coefficients $h_{p,q}\left(f\right)$ are real, constant in *f*, and equal to zero if |p−q|>1. Additionally, the non-zero coefficients $h_{p,q}=h_{p,q}\left(f\right)$ are symmetric in *p* and *q*, i.e., $h_{p,q}=h_{q,p}$, and given by

(29) $$\begin{array}{cc} h_{q,q} & \equiv \pi (N-1),\;\;0\leq q<N\\ h_{q,q-1} & \equiv -\pi q\sqrt{\frac{N^{2}-q^{2}}{4q^{2}-1}},\;\;1\leq q<N. \end{array}$$

This result allows us to write $\phi_{q}^{\prime }\left(f\right)$ as

(30) $$\begin{array}{cc} \phi_{0}^{\prime }\left(f\right) & =jh_{0,0}\phi_{0}\left(f\right)+jh_{1,0}\phi_{1}\left(f\right)\\ \phi_{q}^{\prime }\left(f\right) & =jh_{q-1,q}\phi_{q-1}\left(f\right)+jh_{q,q}\phi_{q}\left(f\right)\\ \; & \;+jh_{q+1,q}\phi_{q+1}\left(f\right),\;\;\;\;\left(0<q<N-1\right)\\ \phi_{N-1}^{\prime }\left(f\right) & =jh_{N-2,N-1}\phi_{N-2}\left(f\right)+jh_{N-1,N-1}\phi_{N-1}\left(f\right). \end{array}$$

### 3.3. Selection of a Frequency Range Using Basis Signatures

For a given truncation index *P*, the span of $a^{\left(0\right)}\left(f\right),$$a^{\left(1\right)}\left(f\right),\;\ldots ,$$a^{\left(P\right)}\left(f\right)$ is the same as the span of $\phi_{0}\left(f\right),$$\phi_{1}\left(f\right),\;\ldots ,$$\phi_{P}\left(f\right)$. Additionally, given a frequency increment Δf, the signature a(f+Δf) can be approximated by the *P*th-order truncated Taylor expansion of a(f+Δf) at Δf=0, i.e.,

(31) $$a\left(f+\Delta f\right)\approx \sum_{p=0}^{P}a^{\left(p\right)}\left(f\right)\frac{{\left(\Delta f\right)}^{p}}{p!},$$

and the accuracy of this formula increases with *P*. These facts imply that projecting onto the span of $\phi_{0}\left(f\right),\;\phi_{1}\left(f\right),\;\ldots ,\;\phi_{P}\left(f\right)$ acts as a filter that selects a range around frequency *f*, i.e., we have that the approximation

(32) $$a\left(f+\Delta f\right)\approx \sum_{p=0}^{P}\phi_{p}\left(f\right)\phi_{p}{\left(f\right)}^{H}a\left(f+\Delta f\right)$$

is accurate in a range whose length increases with *P*. Additionally, if we exclude $\phi_{0}\left(f\right)$ in (32) then the projection selects a punctured range with a null at frequency *f*. To test this filtering effect numerically, let $\rho_{P}\left(\Delta f\right)$ denote the energy of the projection of $\phi_{0}\left(f+\Delta f\right)$ onto the span of $\phi_{1}\left(f\right),\;\phi_{2}\left(f\right),\;\ldots ,\;\phi_{P}\left(f\right)$. $\rho_{P}\left(\Delta f\right)$ can be readily expressed in terms of the real vectors $y_{p}$ in (17):

(33) $$\begin{array}{cc} \rho_{P}\left(\Delta f\right)\equiv \sum_{p=1}^{P}{|\phi_{p}{\left(f\right)}^{H}\phi_{0}\left(f\right)|}^{2}= & \sum_{p=1}^{P}{\left|y_{p}^{T}\mathrm{diag}{\left(a\left(f\right)\right)}^{H}\mathrm{diag}\left(a\left(f+\Delta f\right)\right)y_{0}\right|}^{2}\\ & =\sum_{p=1}^{P}|y_{p}^{T}\mathrm{diag}\left(a\left(\Delta f\right)\right)y_{0}{|}^{2}=\frac{1}{N}\sum_{p=1}^{P}|y_{p}^{T}a\left(\Delta f\right){)|}^{2}. \end{array}$$

**Figure 1:**
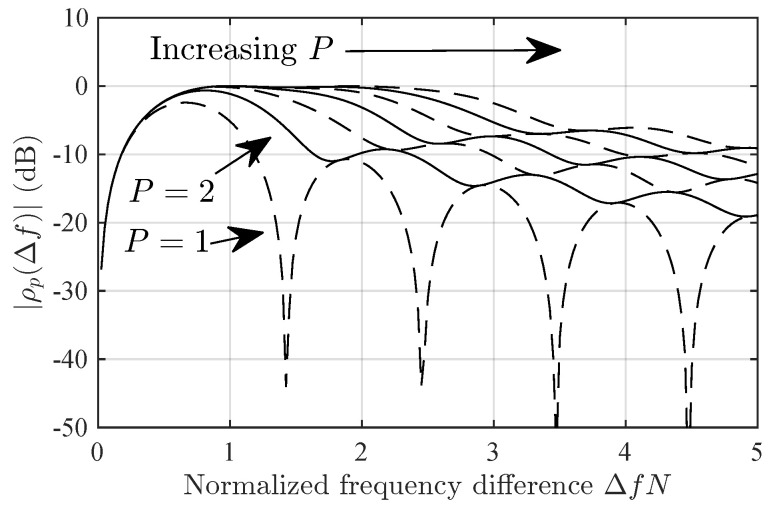
Magnitude of the component of signature $\phi_{0}\left(f+\Delta \right)$ in the span of $\phi_{1}\left(f\right),\;\phi_{2}\left(f\right),\ldots ,\;\phi_{p}\left(f\right)$ as defined in (32).

Figure 1 shows $\rho_{P}\left(\Delta f\right)$ for several values of *P* and N=1024. Note that $\rho_{P}\left(\Delta f\right)$ has a null at Δf=0, i.e., the projection cancels a(f) but, if P≥2, it keeps most of the energy of a(f+Δf) for Δf between, roughly, 0.9/N and a higher value of Δf that increases with *P*. For example, this higher value is, roughly, 1/N for P=3 and 2.2/N for P=7. We can see that we can reduce the energy of $\phi_{0}\left(f+\Delta f\right)$ for significant values of Δf by selecting a small *P*. This filtering effect is useful whenever the periodogram has several peaks of significant amplitude, given that it allows us to separate them by employing a small *P*, i.e., it allows us to process each of them as if it were the only significant peak in the periodogram.

## 4. Statistical Distribution of Correlations with Ortho-Normal Signatures

Consider the single-frequency model in (1) and the corresponding periodogram estimator in (4). Since $\hat{f}$ is the frequency at which P(f;x) attains a maximum, it follows that

(34) $$\frac{\partial }{\partial f}P\left(f;x\right){|}_{f=\hat{f}}=0.$$

Computing this differential from (3), noting that $a\left(f\right)=\sqrt{N}\phi_{0}\left(f\right)$, we have that $\hat{f}$ fulfills the implicit equation

(35) Re{xHϕ0′(f^)ϕ0H(f^)x}=0.

In (1), if we view $s_{o}$ and $f_{o}$ as fixed values and ϵ as a vector of free parameters, we have that x is a function of ϵ,

(36) $$x\left(\epsilon \right)=a\left(f_{o}\right)s_{o}+\epsilon$$

and the same occurs to functions of x such as the periodogram estimate $\hat{f}=\hat{f}\left(\epsilon \right)$ and the correlations

(37) $$\phi_{q}{\left(\hat{f}\right)}^{H}x=\phi_{q}{\left(\hat{f}\left(\epsilon \right)\right)}^{H}x\left(\epsilon \right).$$

Additionally, these functions have known values at ϵ=0, namely, $x=a\left(f_{o}\right)s_{o}$, $\hat{f}=f_{o}$, $\phi_{0}{\left(\hat{f}\right)}^{H}x=s_{o}$ and $\phi_{q}{\left(\hat{f}\right)}^{H}x=0$, q>0. In Appendix B, we derive the first-order Taylor expansions of $\hat{f}\left(\epsilon \right)$ and of the correlations $\phi_{q}{\left(\hat{f}\left(\epsilon \right)\right)}^{H}x\left(\epsilon \right)$ for q>0 but in the phase-corrected form given by

(38) $$\begin{array}{c} {\hat{c}}_{q}\left(\epsilon \right)\equiv e^{-j\hat{\alpha }\left(\epsilon \right)}\phi_{q}{\left(\hat{f}\left(\epsilon \right)\right)}^{H}x\left(\epsilon \right),\;\;q\geq 0, \end{array}$$

where

(39) $$\hat{\alpha }\left(\epsilon \right)\equiv \arg\left\{\phi_{0}{\left(\hat{f}\left(\epsilon \right)\right)}^{H}x\left(\epsilon \right)\right\}.$$

If we define $\alpha_{o}\equiv \arg\left\{s_{o}\right\}$, the resulting expansions are the following:

(40) f^(ϵ)≈fo+Im{e−jαoϕ1(fo)Hϵ}Nh01|so|

(41) c^1(ϵ)≈Re{e−jαoϕ1(fo)Hϵ}

(42) $$\begin{array}{c} {\hat{c}}_{q}\left(\epsilon \right)\approx e^{-j\alpha_{o}}\phi_{q}{\left(f_{o}\right)}^{H}\epsilon ,\;\;q>1. \end{array}$$

For the model in (1), these expansions imply the following approximate distributions:

- $\hat{f}$ is a real Gaussian variable of mean $f_{o}$ and variance
  (43) $$\frac{\sigma^{2}}{2Nh_{10}^{2}{\left|s_{o}\right|}^{2}}=\frac{3\sigma^{2}}{2\pi^{2}{\left|s_{o}\right|}^{2}N\left(N^{2}-1\right)}.$$
  Note that this variance is equal to the Cramer–Rao (CR) bound for single-frequency estimation; (see Equation (17) in [3]).
- The correlations ${\hat{c}}_{q}$, 1≤q≤N−1, are statistically independent.
- ${\hat{c}}_{1}$ is a real Gaussian variable of zero mean and variance $\sigma^{2}/2$.
- ${\hat{c}}_{q}$, q>1, is a complex circularly symmetric Gaussian variable of zero mean and variance $\sigma^{2}$.

## 5. Detection of a Diffuse Component: Spread Factor

The data vector x can be written in terms of the correlations ${\hat{c}}_{q}$ as

(44) $$x=e^{j\hat{\alpha }}({\hat{c}}_{0}{\hat{\phi }}_{0}+\sum_{q=1}^{P}{\hat{c}}_{q}{\hat{\phi }}_{q}+\sum_{q=P+1}^{N-1}{\hat{c}}_{q}{\hat{\phi }}_{q})$$

where, in short form, we write ${\hat{c}}_{q}\left(\epsilon \right)$ and $\phi_{q}\left(\hat{f}\left(\epsilon \right)\right)$ as ${\hat{c}}_{q}$ and ${\hat{\phi }}_{q}$, respectively. We consider that the first summation may be affected by a diffuse signal component, while the second is mostly produced by noise. The choice of *P* depends on the assumptions on the frequency distribution s(f) in (5) and also on the filtering effect discussed in Section 3.3. The detection task consists of deciding between the single component model in (1) (H0 hypothesis) and the diffuse component model in (5) (H1 hypothesis). Under H0, the distribution of the correlations ${\hat{c}}_{q}$, q=1,2,…,P is that derived in the previous section and from it we can deduce that the sum

(45) $$\frac{2}{\sigma^{2}}\sum_{q=1}^{P}{\hat{c}}_{q}$$

follows a Chi-Square distribution of order 2P−1. Additionally, we can determine a threshold $\gamma_{P}$ for which the condition

(46) $$\frac{2}{\sigma^{2}}\sum_{q=1}^{P}{\hat{c}}_{q}>\gamma_{P}$$

implies the presence of a signal component for a given false-alarm probability $P_{\mathrm{FA},P}$. Finally, if H1 is accepted, then a measure of the “diffuseness” of the periodogram peak is the ratio between the estimated signal components energies in the spans of ${\hat{\phi }}_{1},\;,{\hat{\phi }}_{2},\;\;\ldots ,$ ${\hat{\phi }}_{P}$ and ${\hat{\phi }}_{0}$, i.e.,

(47) $$\hat{D}\equiv \frac{1}{|{\hat{c}}_{0}{|}^{2}-\sigma^{2}}\sum_{q=1}^{P}{\left|{\hat{c}}_{q}\right|}^{2}-\sigma^{2}\left(1-\delta_{q}/2\right).$$

We call $\hat{D}$ the spread factor. Additionally, we define $\hat{D}=0$ if H1 is rejected.

## 6. Computation of Correlations from DFT Samples

The periodogram estimate $\hat{f}$ and the proposed detector and estimator require in their computation the value of one or more correlations of either the form $a^{\left(p\right)}{\left(f\right)}^{H}x$ or $\phi_{p}{\left(f\right)}^{H}x$ at arbitrary frequencies *f*. These correlations involve summations with a large number of summands *N*, namely,

(48) $$\begin{array}{cc} a^{\left(p\right)}{\left(f\right)}^{H}x & =\sum_{n=0}^{N-1}{\left(j2\pi n\right)}^{p}{\left[x\right]}_{n+1}e^{-j2\pi nf}, \end{array}$$

(49) $$\begin{array}{cc} \phi_{p}{\left(f\right)}^{H}x & =\sum_{n=0}^{N-1}y_{p}\left(n\right){\left[x\right]}_{n+1}e^{-j2\pi nf} \end{array}$$

for p=0,1,…, *P*. Thus, we may expect a large computational burden if the summations in either (48) or (49) are directly evaluated for any *p*, 0≤p≤P. However, the fact is that all the correlations in (48) and (49) can be obtained with small complexity from just of a few of the DFT samples in (13). Actually, as we show in the sequel, it is possible to obtain them from 2Q+1 samples of the DFT using a so-called barycentric interpolator [18], where Q>0 is an integer that controls the interpolation accuracy. Specifically, the interpolation error decreases exponentially with *Q* and, in practice, a small *Q* is sufficient (typically, *Q* ranges from 3 to 6). The method for doing this is based on the following ideas:

**(1)** Note that the correlations $\phi_{p}{\left(f\right)}^{H}x$ can be computed from the correlations $a^{\left(p\right)}{\left(f\right)}^{H}x$ due to (27), i.e.,

(50) $$\phi_{p}{\left(f\right)}^{H}x=\sum_{q=0}^{p}\frac{\psi_{p,q}}{{\left(-j2\pi \right)}^{q}}a^{\left(q\right)}{\left(f\right)}^{H}x,\;\;p=0,\;1,\;\;\ldots ,\;P.$$

So, it is only necessary to compute the correlations of the first type $a^{\left(p\right)}{\left(f\right)}^{H}x$, p=0,1,…, *P*.

**(2)** The initial correlation $a{\left(f\right)}^{H}x$ can be written as

(51) $$a{\left(f\right)}^{H}x=e^{-j\pi Nf}\eta \left(bNf\right)$$

where

(52) $$\eta \left(u\right)\equiv \sum_{p=-N/2}^{N/2-1}{\left[x\right]}_{n+N/2+1}e^{-j2\pi nu/(bN)}.$$

Additionally, any derivative $a^{\left(p\right)}{\left(f\right)}^{H}x$ can be obtained form the derivatives of η(u) of orders 0 to *P* through Leibnitz’s formula for the *p*th derivative of a product; i.e., the *p*th order derivative of (51) is

(53) a(p)(f)Hx=∑k=0ppk(−jπN)p−ke−jπNf(bN)kη(k)(bNf)=e−jπNf(−jπN)p∑k=0ppkjbπkη(k)(bNf).

Therefore, the computation of the correlations $a^{\left(p\right)}{\left(f\right)}^{H}x$ can be reduced to the computation of the derivatives $\eta^{\left(p\right)}\left(u\right)$ for arbitrary *u* and p=0,1,…,P.

**(3)** η(u) is a band-limited signal in *u* of two-sided bandwidth 1/b<1 and, as a consequence, it can be computed from its samples with spacing 1 using the sinc series. More precisely, let *n* and *v* be defined by the modulo-1 decomposition of *u*, given by

u=n+vwithintegernand−1/2≤v<1/2.

The sinc series in *v* for the signal η(n+v) is

(54) $$\eta \left(u\right)=\sum_{q=-\infty }^{\infty }\eta \left(n+q\right)\mathrm{sinc}\left(v-q\right).$$

Note that, from (51), η(n+q) is equal to an exponential multiplied by $a{\left(\left(n+q\right)/\left(bN\right)\right)}^{H}x$ and this last value is one of the DFT samples in (13). Therefore, all the samples η(n+q) appearing in (54) are already available.

**(4)** Since there is oversampling in (54), this series is also valid for a product η(v)w(v) where w(v) is a fixed signal whose two-sided bandwidth is below the limit 1−1/b. So we have

(55) $$\eta \left(u\right)w\left(v\right)=\sum_{q=-\infty }^{\infty }\eta \left(n+q\right)w\left(q\right)\mathrm{sinc}\left(v-q\right).$$

Now, if $w\left(v\right)=g_{Q}\left(v\right)$ where $g_{Q}\left(v\right)$ is a fixed pulse whose tails are negligible outside the *v* range [−Q,Q] for a given truncation index *Q*, then we may truncate (55) at ±Q and extract the function sin(v−q) implicit in sinc(v−q), i.e.,

(56) $$\begin{array}{cc} \eta \left(v\right)g_{Q}\left(v\right) & \approx \sum_{q=-Q}^{Q}\eta \left(n+q\right)g_{Q}\left(q\right)\mathrm{sinc}\left(v-q\right)\approx \frac{\sin(\pi v)}{\pi }\sum_{q=-Q}^{Q}\frac{{\left(-1\right)}^{q}\eta \left(n+q\right)g_{Q}\left(q\right)}{v-q}. \end{array}$$

Additionally, (55) and (56) also hold if η(v) is replaced by 1 (which has zero bandwidth). So, we also have the approximation

(57) $$g_{Q}\left(v\right)\approx \frac{\sin(\pi v)}{\pi }\sum_{q=-Q}^{Q}\frac{{\left(-1\right)}^{q}g_{Q}\left(q\right)}{v-q}.$$

Dividing (56) by (57), we obtain an approximation for η(v),

(58) $$\eta \left(v\right)\approx \sum_{q=-Q}^{Q}\frac{{\left(-1\right)}^{q}\eta \left(n+q\right)g_{Q}\left(q\right)}{v-q}/\sum_{q=-Q}^{Q}\frac{{\left(-1\right)}^{q}g_{Q}\left(q\right)}{v-q}.$$

This is a so-called barycentric interpolation formula, and we can readily see that it only involves arithmetic operations, given that the values $g_{Q}\left(q\right)$ are constant in *u* and can be pre-computed. Additionally, (58) can be extremely accurate, as shown in [18], for a proper choice of $g_{Q}\left(v\right)$. Actually, for Knab’s pulse [25] given by

(59) $$g_{Q}\left(v\right)\equiv \frac{\mathrm{sinc}\left(\left(1-1/b\right)\sqrt{v^{2}-{\left(Q+1\right)}^{2}}\right)}{\mathrm{sinc}\left(j(1-1/b)(Q+1)\right)}$$

the error in (58) is bounded by

(60) $$\frac{A}{\sinh(\pi (1-1/b)Q)}$$

where *A* is the maximum of η(u) for *u* in [0,1]. Note that this last bound implies that the error decreases as $e^{-\pi (1-1/b)Q}$ and, therefore, a small *Q* ensures high accuracy. Furthermore, the derivative of (58) up to any order can be computed using the Horner-like scheme described in ([18] Sec. IV). The complexity of obtaining $\eta^{\left(p\right)}\left(u\right)$ for p=0,1,…,P is O(QP), i.e., independent of the correlation length *N*.

From these ideas, we can readily deduce a method to interpolate (48) and (49) consisting of the following steps:

1. Compute $\eta^{\left(p\right)}\left(v\right)$, p=0,1,…,P by evaluating the barycentric interpolator in (58).
2. Compute $a^{\left(p\right)}{\left(f\right)}^{H}x$ from $\eta^{\left(p\right)}\left(v\right)$, p=0,1,…,P, using (53).
3. Compute $\phi_{p}{\left(f\right)}^{H}x$ from $a^{\left(p\right)}{\left(f\right)}^{H}x$, p=0,1,…,P, using (50).

## 7. Numerical Assessment

We consider the periodogram with N=1024 and scenarios of one of the following two types:

- **Type 1.** Scenario with two frequency components of unequal power. Its distribution s(f) in (5) is
  (61) $$s\left(f\right)=a_{1}\delta \left(f-f_{1}\right)+a_{2}\delta \left(f-f_{1}-\Delta f\right)$$
  where $|a_{1}\left|>\right|a_{2}|$ and Δf>0.
- **Type 2.** Scenario with main frequency and a diffuse component. Its distribution is
  (62) $$s\left(f\right)=a_{1}\delta \left(f-f_{1}\right)+\sum_{q=2}^{11}a_{q}\delta \left(f-f_{q}\right),$$
  where the summation models the diffuse component and $f_{2},\;f_{3},\;\ldots ,\;f_{11}$ lie in a range [−Δf,Δf], Δf>0.

For both types, the power of the second component is set 5 dB below that of the first component. The signal-to-noise ratio (SNR) is defined as the ratio between the first component’s power and $\sigma^{2}$. In scenarios in which the SNR is not variable, it is set to 0 dB.

The specific scenarios in the numerical assessment are the following:

- **Delta.** Type-1 scenario with $f_{1}=0.345$, Δf=0.61/N, and $\arg\{a_{2}/a_{1}\}$ uniformly distributed in [0,2π].
- **Delta-θ.** The same as Delta, but viewing the phase of $a_{2}$ relative to $a_{1}$ as a variable $\theta \equiv \arg\{a_{2}/a_{1}\}$.
- **Delta-Δf.** The same as Delta, but with variable Δf.
- **Diffuse.** Type-2 scenario with Δf=0.61/N and amplitudes, phases, and frequencies in summation following uniform distributions in [0,1], [0,2π], and [0.345−Δf,0.345+Δf], respectively.
- **Diffuse-Δf.** The same as Diffuse, but with variable Δf.

We consider the following two detectors:

- **Secondary peak (SP).** The second signal component is detected if the residual periodogram given by
  (63) $$P\left(f;x,\hat{f}\right)\equiv {\left|a{\left(f\right)}^{H}\left(I_{N}-\phi_{0}\left(\hat{f}\right)\phi_{0}{\left(\hat{f}\right)}^{H}\right)x\right|}^{2}$$
  has a peak above a detection threshold with 0.05 false-alarm probability.
- **Proposed (Prop-P).** The detector in Section 5 with a given truncation order *P* and 0.05 false-alarm probability.

### 7.1. Performance Versus Signal-to-Noise Ratio

**Figure 2:**
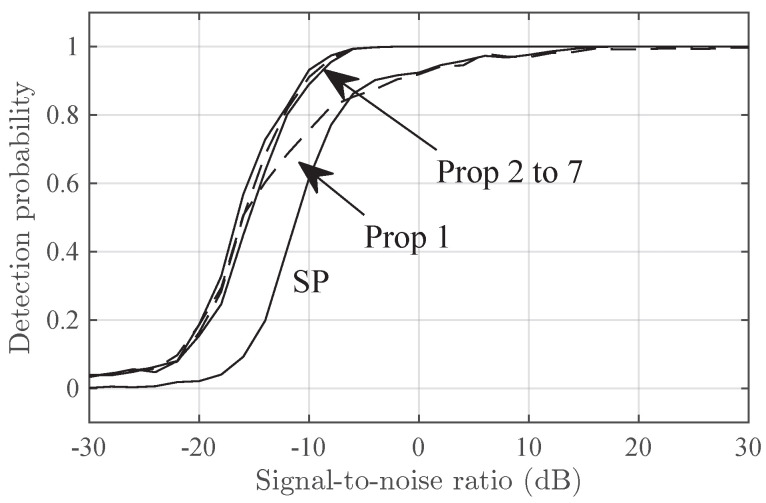
The detection probability in the Delta-2 scenario versus SNR for the SP and Prop-1 to Prop-7 detectors.

**Figure 3:**
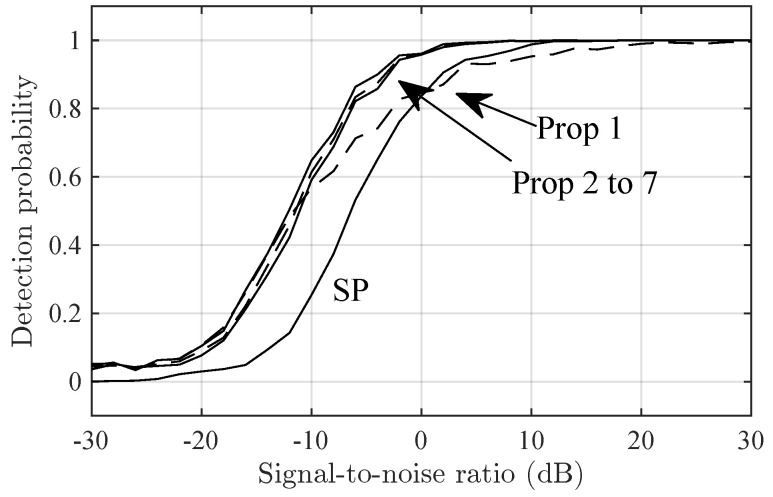
The detection probability in the Diffuse-2 scenario versus SNR for the SP and Prop-1 to Prop-7 detectors.

Figure 2 and Figure 3 show the detection probability versus the SNR in the Delta and Diffuse scenarios, respectively. In both figures, Prop-2 to Prop-7 have similar performance and outperform SP by, roughly, 6 dB in SNR. Prop-1 has a intermediate performance between that of SP and Prop-2 to Prop-7.

**Figure 4:**
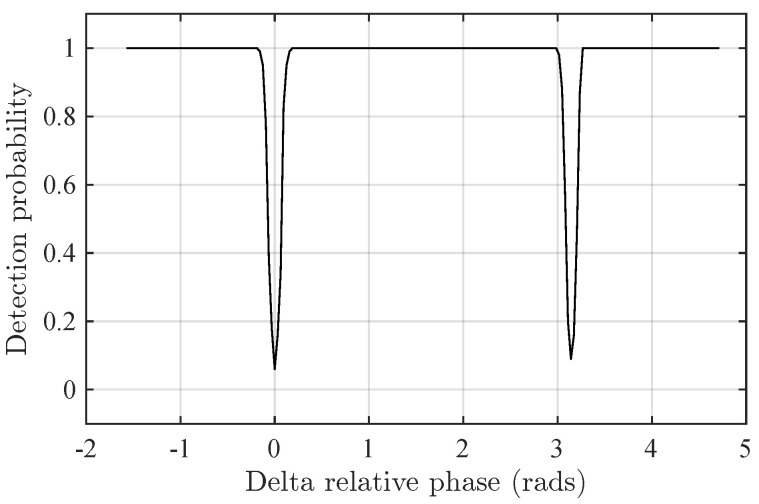
Detection probability of Prop-1 detector in the Delta-θ scenario versus the relative phase θ.

In order to assess the behavior of Prop-1, Figure 4 shows the detection probability of Prop-1 versus the relative phase θ in Delta-θ. Note that the detection performance of Prop-1 drops at θ=0,π, i.e., Prop-1 is unable to detect the second component for these phases. This seems to be a consequence of the fact that $c_{1}\left(\hat{f}\right)$ is real.

### 7.2. Detection Performance Versus Frequency Spread

**Figure 5:**
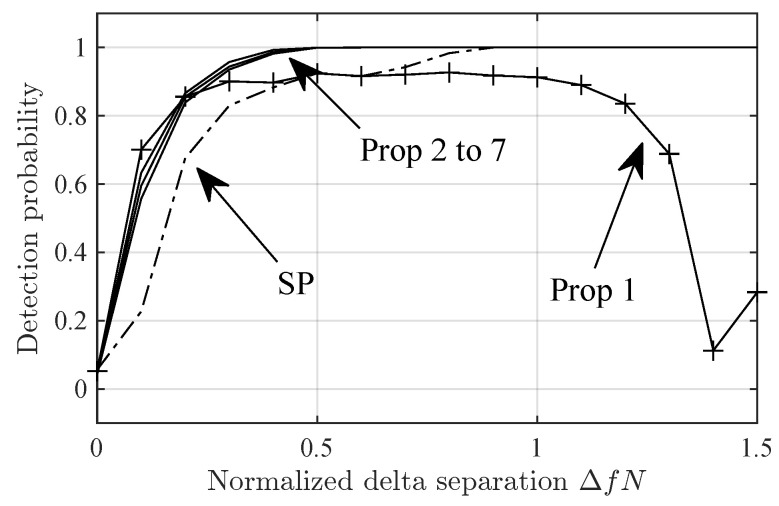
Detection probability for the SP and Prop-1 to -7 detectors in the Delta-Δf scenario versus ΔfN.

**Figure 6:**
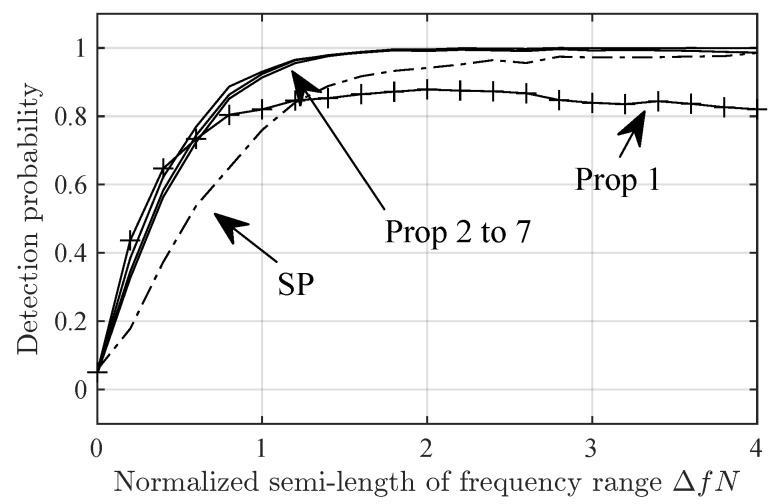
Detection probability for the SP and Prop-1 to -7 detectors in the Diffuse-Δf scenario versus the frequency separation.

Figure 5 and Figure 6 show the detection probability in the Delta-Δf and Diffuse-Δf scenarios versus Δf. Note that Prop-1 fails in Delta-2 scenario at ΔfN≈0.4. This is due to the zero at that position in $\rho_{1}\left(\Delta f\right)$, (Figure 1). However, this is not an issue if P>1, given that then $\rho_{P}\left(\Delta f\right)$ has no nulls for Δf>0. The null in $\rho_{1}\left(\Delta f\right)$ seems to be the cause for the sub-optimal detection probability in Diffuse-Δf for high ΔfN.

### 7.3. Spread Factor

**Figure 7:**
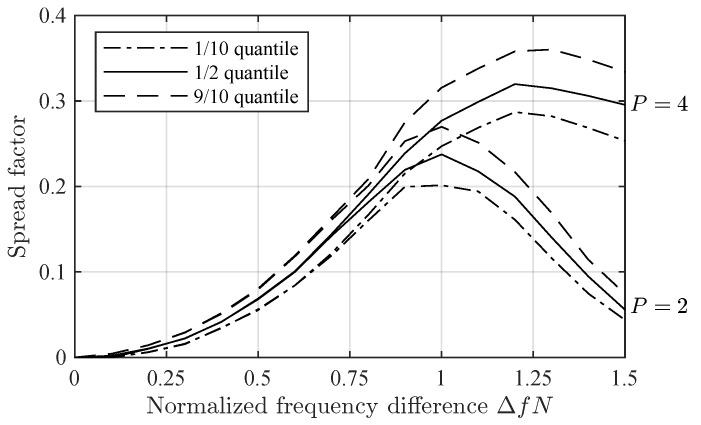
Spread factor for P=2 and P=4 in Delta-Δf scenario, expressed using the 1/10, 1/2 and 9/10 quantiles, versus ΔfN.

Figure 7 shows the spread factor in Delta-Δf scenario versus ΔfN. Note that, for small ΔfN, the spread factors for Prop-2 and Prop-4 are roughly the same. However, for high ΔfN, Prop-2 estimates a much lower spread factor. This seems to be due to the drop in $\rho_{2}\left(\Delta f\right)$ for ΔfN roughly greater than 0.9 (Figure 1).

## 8. Conclusions

We have presented a detector for a diffuse component in any periodogram peak of significant amplitude and an estimator of the spread of such component. Basically, the detector tests whether the power in the span of a number of the signature’s derivatives is too high for a noise realization, where the derivatives are evaluated at the peak’s frequency. Then, the “spreadness” of the diffuse component is estimated through the so-called spread factor, which is the ratio between the estimated signal power in the span of the signature and the estimated signal power in the derivatives’ span. Both the detector and the estimator exploit the Vandermonde structure of the frequency signature through the properties of discrete Chebyshev polynomials. We have assessed their performance numerically.

## Data Availability

Data are contained within the article.

## Appendix A. Proof of (29)

Define the N×N diagonal matrix ${\left[\Lambda \right]}_{n}\equiv n-1$, n=1,2,…,N, and let the subscript ${\left(\cdot \right)}_{1}$ denote multiplication by *n* on any polynomial $z\left(n\right)\in \pi_{N-1}$, i.e., $z_{1}\left(n\right)=nz\left(n\right)$.

Recalling (17), we see that $\phi_{q}^{\prime }\left(f\right)$ can be written as

(A1) $$\phi_{q}^{\prime }\left(f\right)=j2\pi \Lambda \mathrm{diag}\left(a\left(f\right)\right)y_{q}.$$

Next, left multiply (28) by $-j\phi_{p}{\left(f\right)}^{H}$ and use (17), the last formula, and (21):

(A2) $$\begin{array}{c} h_{p,q}\left(f\right)=-j\phi_{p}{\left(f\right)}^{H}\phi_{q}^{\prime }\left(f\right)=y_{p}^{T}\mathrm{diag}\left(a\left(-f\right)\right)2\pi \Lambda \mathrm{diag}\left(a\left(f\right)\right)\;y_{q}\\ \;=2\pi y_{p}^{T}\Lambda y_{q}=2\pi \langle y_{p},y_{q,1}\rangle =\frac{2\pi }{∥\alpha_{p}∥∥\alpha_{q}∥}\langle \alpha_{p},\alpha_{q,1}\rangle . \end{array}$$

So we see that $h_{p,q}\left(f\right)$ is a real constant $h_{p,q}$. $h_{0,0}$ can be directly computed using the inner product definition in (18). The result is

(A3) $$h_{0,0}=\pi \left(N-1\right).$$

Next, to determine the rest of coefficients $h_{p,q}$, let us write the last inner product in (A2) in terms of the recurrence formula in (20). If we replace *p* with q+1 in (20) and then solve for $n\alpha_{q}\left(n\right)$, we obtain, after some operations,

(A4) $$\alpha_{q,1}\left(n\right)=-\frac{N^{2}-q^{2}}{4q+2}\alpha_{q-1}\left(n\right)+\frac{N-1}{2}\alpha_{q}\left(n\right)-\frac{{\left(1+q\right)}^{2}}{4q+2}\alpha_{q+1}\left(n\right).$$

And forming the inner product with $\alpha_{p}$, we obtain

(A5) $$\langle \alpha_{p},\alpha_{q,1}\rangle =-\frac{N^{2}-q^{2}}{4q+2}∥\alpha_{q-1}{∥}^{2}\delta_{q-1-p}+\frac{N-1}{2}∥\alpha_{q}{∥}^{2}\delta_{q-p}-\frac{{\left(q+1\right)}^{2}}{4q+2}{∥\alpha_{q+1}∥}^{2}\delta_{q+1-p}.$$

We may derive from this formula several results. First, it is $h_{p,q}=0$ if |p−q|>1. Second, taking p=q and substituting the result into (A2), we obtain

(A6) $$h_{q,q}=\pi \left(N-1\right),\;\;q>0.$$

Third, note that we may exchange *p* and *q* in $\langle \alpha_{p},\alpha_{q,1}\rangle$, given that

(A7) $$\langle \alpha_{p},\alpha_{q,1}\rangle =\sum_{n=0}^{N-1}\alpha_{p}\left(n\right)\left(n\alpha_{q}\left(n\right)\right)=\sum_{n=0}^{N-1}\alpha_{q}\left(n\right)\left(n\alpha_{p}\left(n\right)\right)=\langle \alpha_{q},\alpha_{p,1}\rangle .$$

Therefore, $h_{p,q}=h_{q,p}$ for any p,q≥0. And fourth, exchange *p* and *q* on the right-hand side of (A5) and then write any expression multiplying a Dirac delta in terms of *q* only:

(A8) $$\begin{array}{c} \langle \alpha_{p},\alpha_{q,1}\rangle =-\frac{N^{2}-p^{2}}{4p+2}∥\alpha_{p-1}{∥}^{2}\delta_{p-1-q}+\frac{N-1}{2}∥\alpha_{p}{∥}^{2}\delta_{p-q}-\frac{{\left(p+1\right)}^{2}}{4p+2}{∥\alpha_{p+1}∥}^{2}\delta_{p+1-q}\\ =-\frac{N^{2}-{\left(q+1\right)}^{2}}{4(q+1)+2}∥\alpha_{q}{∥}^{2}\delta_{p-1-q}+\frac{N-1}{2}∥\alpha_{q}{∥}^{2}\delta_{p-q}-\frac{q^{2}}{4(q-1)+2}{∥\alpha_{q}∥}^{2}\delta_{q+1-p}. \end{array}$$

Equating the coefficient of $\delta_{q+1-p}$ in (A5) and (A8), we obtain

(A9) $$-\frac{N^{2}-q^{2}}{4q+2}∥\alpha_{q-1}{∥}^{2}=-\frac{q^{2}}{4q-2}{∥\alpha_{q}∥}^{2},$$

which can be written as

(A10) $$\frac{∥\alpha_{q}{∥}^{2}}{∥\alpha_{q-1}{∥}^{2}}=\frac{N^{2}-q^{2}}{q^{2}}\cdot \frac{2q-1}{2q+1}.$$

So, from (A2), (A5) and (A10) with p=q−1, we have

(A11) $$\begin{array}{cc} h_{q-1,q} & =\frac{2\pi \langle \alpha_{q-1},\alpha_{q,1}\rangle }{∥\alpha_{q-1}∥∥\alpha_{q}∥}=-2\pi \frac{N^{2}-q^{2}}{4q+2}\frac{∥\alpha_{q-1}∥}{∥\alpha_{q}∥}=-\pi \frac{N^{2}-q^{2}}{2q+1}\sqrt{\frac{q^{2}\left(2q+1\right)}{\left(N^{2}-q^{2}\right)\left(2q-1\right)}}\\ & =-\pi q\sqrt{\frac{N^{2}-q^{2}}{4q^{2}-1}}. \end{array}$$

## Appendix B. Perturbation Analysis of the Periodogram Estimator

To simplify the derivations in this appendix, we let *y* denote any of the real or imaginary parts of the components of ϵ, i.e., the parts

(A12) Re{[ϵ]n},Im{[ϵ]n},n=1,2,…,N.

In the sub-sections that follow, we first compute the derivatives in *y* and then identify *y* with each of the parts in (A12) in order to obtain total derivatives. We additionally adopt the following simplified notation:

- $\hat{f}$, x and ${\hat{\phi }}_{q}$ denote $\hat{f}\left(\epsilon \right)$, x(ϵ) and $\phi_{q}\left(\hat{f}\left(\epsilon \right)\right)$, respectively.
- $\phi_{qo}$ stands for $\phi_{q}\left(f_{o}\right)$.
- The subscript “*y*” stands for the derivative in *y*.
- Subscript “0” denotes evaluation at ϵ=0; for example $\epsilon_{y0}$ is the derivative in *y* of ϵ at ϵ=0.

### Appendix B.1. Total Derivative of Periodogram Estimate $\hat{f}$(**ϵ**)

Substituting the first formula in (30) into (35), taking into account that $h_{00}$ and $h_{10}$ are real and $h_{10}\neq 0$, we have

Re{xH(jh00ϕ^0+jh10ϕ^1)ϕ^0Hx}=0Re{jh00|xHϕ^0|2+jh10xHϕ^1ϕ^0Hx}=0Im{xHϕ^1ϕ^0Hx}=0.

Let us differentiate this equation in *y*. We have

0=Im{ϵyHϕ^1ϕ^0Hx+xH(ϕ^1ϕ^0H)yx+xHϕ^1ϕ^0Hϵy}=Im{ϵyHϕ^1ϕ^0Hx+xH(ϕ^1′ϕ^0H+ϕ^1ϕ^0′H)xf^y+xHϕ^1ϕ^0Hϵy}=Im{ϵyHϕ^1ϕ^0Hx+xHϕ^1′ϕ^0Hxf^y+xHϕ^1ϕ^0′Hxf^y+xHϕ^1ϕ^0Hϵy}.

Next, let us set ϵ=0. Then, ${\hat{\phi }}_{1}=\phi_{1o}$, ${\hat{\phi }}_{0}^{H}x=\sqrt{N}s_{o}$, $x^{H}{\hat{\phi }}_{1}^{\prime }=j\sqrt{N}h_{01}s_{o}^{*}$, and $x^{H}{\hat{\phi }}_{1}=0$. So we have

0=Im{ϵy0H·ϕ1o·Nso+jNh01so*·Nso·f^y0}0=Im{Nϵy0Hϕ1oso+jNh01|so|2f^y0}0=−Im{so*ϕ1oHϵy0}+Nh01|so|2f^y00=−Im{e−jαoϕ1oHϵy0}+Nh01|so|f^y0

And solving for ${\hat{f}}_{y0}$, we obtain

f^y0=Im{e−jαoϕ1oHϵy0}Nh01|so|.

This expression is linear in the parts specified in (A12) and, therefore, identifying *y* with each of them, we obtain the total derivative of $\hat{f}$ at ϵ=0,

df^ϵ=0(γ)=Im{e−jαoϕ1oHγ}Nh01|so|.

This formula directly gives (40).

### Appendix B.2. Total Derivatives of Correlations ${\hat{c}}_{q}$(**ϵ**)

Let us differentiate (38) in *y*. We have

$${\hat{c}}_{qy}={\left(e^{-j\hat{\alpha }}{\hat{\phi }}_{q}^{H}x\right)}_{y}={\left(e^{-j\hat{\alpha }}\right)}_{y}{\hat{\phi }}_{q}^{H}x+e^{-j\hat{\alpha }}\left({\hat{\phi }}_{q}^{\prime H}x{\hat{f}}_{y}+{\hat{\phi }}_{q}^{H}\epsilon_{y}\right).$$

If ϵ=0 then ${\hat{\phi }}_{q}=\phi_{qo}$, ${\hat{\phi }}_{q}^{H}x=0$, $e^{-j\hat{\alpha }}=e^{-j\alpha_{o}}$, $x=\sqrt{N}\phi_{0o}s_{o}$, and ${\hat{\phi }}_{q}^{\prime H}x=-j\sqrt{N}h_{0,q}s_{o}$. So, substituting (A13), we have

(A13) c^qyo=e−jαo−jh0,qIm{e−jαoϕ1oHϵy0}h01|so|+ϕqoHϵy0=−jh0,qIm{e−jαoϕ1oHϵy0}h01+e−jαoϕqoHϵy0=Re{e−jαoϕqoHϵy0}+j1−h0,qh01Im{e−jαoϕqoHϵy0}=Re{e−jαoϕqoHϵy0}+j1−δq−1Im{e−jαoϕqoHϵy0}.

In the last line, we have used the fact that $h_{p,q}=0$ if |p−q|>1. Again, identifying *y* with any of the parts in (A12), we obtain the total derivatives

(A14) dc^1,ϵ=0(γ)=Re{e−jαoϕqoHγ},

and, for q>0,

(A15) $$\begin{array}{c} d\;{\hat{c}}_{q,\epsilon =0}\left(\gamma \right)=e^{-j\alpha_{o}}\phi_{qo}^{H}\gamma . \end{array}$$

These two formulas give (41) and (42).
